# HCV Defective Genomes Promote Persistent Infection by Modulating the Viral Life Cycle

**DOI:** 10.3389/fmicb.2018.02942

**Published:** 2018-12-03

**Authors:** Eirini Karamichali, Hajar Chihab, Athanassios Kakkanas, Agnes Marchio, Timokratis Karamitros, Vasiliki Pogka, Agoritsa Varaklioti, Antonis Kalliaropoulos, Beatrice Martinez-Gonzales, Pelagia Foka, Ioannis Koskinas, Andreas Mentis, Soumaya Benjelloun, Pascal Pineau, Urania Georgopoulou

**Affiliations:** ^1^Molecular Virology Laboratory, Hellenic Pasteur Institute, Athens, Greece; ^2^Virology Unit, Viral Hepatitis Laboratory, Institut Pasteur du Maroc, Casablanca, Morocco; ^3^Institut Pasteur, INSERM U993, Unité “Organisation Nucléaire et Oncogenèse”, Paris, France; ^4^Medical Microbiology Laboratory, Hellenic Pasteur Institute, Athens, Greece; ^5^Blood Center and National Centre for Congenital Bleeding Disorders, Laiko General Hospital, Athens, Greece; ^6^2nd Department of Internal Medicine, Medical School of Athens, Hippokration General Hospital, Athens, Greece

**Keywords:** hepatitis C, defective genomes, exosomes, viral replication, viral persistence

## Abstract

Defective interfering (DI) RNAs have been detected in several human viruses. HCV in-frame deletions mutants (IFDMs), missing mainly the envelope proteins, have been found in patient sera and liver tissues. IFDMs replicate independently and can be *trans*-packaged into infectious virions in the presence of full length viral genome. So far, their biological role is unclear. In this study, we have isolated and cloned IFDMs from sera samples and liver tissues of patients infected with HCV genotypes 1b, 2a, and 3a. IFDMs were present in up to 26% of samples tested. Using the *in vitro* HCV cell culture system, co-expression of the wild type (wt) HCV replicon with HCV IFDMs RNA resulted in increased HCV replication. Additionally, co-transfection of the HCV full length genome RNA and a defective mutant missing the envelope region led to increased viral release, collectively suggesting an important biological role for IFDMs in the virus life cycle. Recently, exosomes, masters of intercellular communication, have been implicated in the transport of HCV viral genomes. We report for the first time that exosomal RNA isolated from HCV sera samples contains HCV defective genomes. We also demonstrate that inhibition of exosomal biogenesis and release influences HCV viral replication. Overall, we provide evidence that the presence of HCV IFDMs affects both viral replication and release. IFDMs exploit exosomes as means of transport, a way to evade the immune system, to spread more efficiently and possibly maintain persistent infection.

## Introduction

Defective interfering RNAs originating from a viral genome, generate DI viral particles. DI viruses can replicate, but cannot be packaged into viral particles unless the infectious wt helper virus provides the necessary complementary function(s). DI genomes have been identified in different human viruses, such as dengue virus, hepatitis B (Rosmorduc et al., 1995), hepatitis C (HCV) (Cheroni et al., 2015). hepatitis A (Nuesch, 1989) and influenza A viruses (Dimmock and Easton, 2014; Laske et al., 2016).

The HCV deletion mutants reported in sera or liver biopsies of patients with chronic hepatitis C (Bernardin et al., 2007; Noppornpanth et al., 2007; Sugiyama et al., 2009; Ohtsuru et al., 2013), resemble the DI particles described for many viruses, including members of the *Flaviviridae* family, such as tick-borne encephalitis, Murray Valley encephalitis, West Nile, and Japanese encephalitis viruses (Brinton, 1983; Lancaster et al., 1998; Mandl et al., 1998; Aaskov et al., 2006; Yoon et al., 2006). These natural HCV defective genomes usually coexist with the full length wt and have been found to contain large in-frame deletions of sequences that code for E1 up to NS2 viral proteins. Different studies confirm that HCV defective genomes preserve viral regions that express the structural core protein and the non-structural proteins involved in RNA replication (Yagi et al., 2005; Bernardin et al., 2007; Noppornpanth et al., 2007). Pacini et al. (2009) demonstrated that the natural deletions are replication-competent and they are *trans*-packaged into infectious virions when co-expressed together with wt virus. It has been suggested that the long-term, stable detection of HCV subgenomes reported in plasma, could reflect the occasional generation of intracellular replicons. Any deleted structural features, including envelope glycoproteins, could be provided by *trans*-complementation in cells co-infected with wt helper HCV. Then, the subgenome-containing particles released into the bloodstream from co-infected cells would be competent for cell entry and further RNA replication into new target cells. The resulting sustained amplification of these truncated genomes could account for the fact that they are persistently observed in patient plasma, in high levels (Bernardin et al., 2007).

The biological role of DI genomes in viral evolution has not been elucidated yet. In particular, the relation of DIs with various viral processes, the immune system, pathogenesis, and viral transmission are currently unclear. Additionally, whether the presence of DIs leads to the establishment of a persistent infection or viral clearance remains an open question. Thus, further research in this area is considered necessary, as it could lead to improvements in both prediction of clinical outcomes and patient treatment (Sun et al., 2015).

In this study, we seek to assign a biological role(s) to the HCV in frame deletion mutants (IFDMs), by investigating their input in viral replication and release. Furthermore, we aim to delineate the host cellular mechanisms that IFDMs exploit in order to fulfil their putative biological purpose in HCV persistence.

## Materials and Methods

### Materials

Chronic HCV sera samples (genotypes 3a, 1b, and 2a) were obtained from the Departments of Medical Microbiology of Hellenic Pasteur Institute, the 2nd Department of Internal Medicine, Hippokration Hospital, Medical School of Athens and Pasteur du Maroc. The liver tissue samples of HCV (genotype 1b) were collected at Pasteur Paris. Healthy blood donors were used as control. This study has been approved by the Ethics Committee of Hippokration Hospital of Athens, the Bioethics Committee of Pasteur Institute of Maroc and the Bioethics Committee of Institute Pasteur Paris. All the subjects signed a written informed consent in accordance with the declaration of Helsinki. All of them have already been included in various studies published in different journals. Sample numbers and genotypes of the sera and liver tissues used in this study are presented in Table 1.

**Table 1:** % prevalence of IFDMs detected in sera samples infected with HCV genotypes 3a, 2a, 1b **(A)** and liver tissue infected with HCV genotypes 1b, 2a **(B)**.

HCV genotype	(*n*)	WT (*n*)	NONA (*n*)	IFDMs (*n*)	% IFDMs
**A**					
1b	23	8	9	6	26
2a	35	9	19	7	20
3a/c	31	9	14	8	25,8
**B**					
2a	12 (4^∗^)		7	1	8,3
1b	45	32	4	9	20

*NONA, non-amplified; (*n*), number of samples. ^∗^mispriming (checked by sequence).*

### Viral RNA Isolation, PCR and Plasmids

For all genotypes, HCV RNA from sera samples was isolated with the viral RNA isolation kit (Macherey – Nagel) according to the manufacturer’s instructions. Degenerate primers specific for genotypes 3a, 1b, and 2a were designed based on sequences downloaded from Los Alamos HCV sequence database. Specifically for genotype 3a, RT-PCR was performed with specific primer 5′-TAC ATT TGG AGC GCR GGA TGT TTG-3′ using the PrimeScript Reverse Transcriptase (TakaRa). cDNA fragments were amplified by nested PCR using LA Taq polymerase (TakaRa) and two different pairs of primers 1st forward: 5′CTG TGA GGA ACT ACT GTC TTC ACG C3′, 1st reverse: 5′-CCY GCA CCA TGR TAA ACA GTC CA-3′, 2nd Forward: 5′-CTA GCC GAG TAG CGT TGG G-3′ 2nd Reverse: 5′-CCR GTC ACC ACG TTC TTR TCC CTG CC-3′. For genotypes 1b and 2a, cDNA was synthesized using the High Capacity cDNA Archive Kit (Perkin Elmer) with RT primers 5′-GTT TCC ATR GAY TCR ACR GG-3′and 5′-AGC CGG GAT GAC RTC AGC GTT-3′ Then, a nested PCR was performed using MyFi Mix (Bioline) and specific primers 1st forward 5′-ATG AGT GTC GTA CAG CCT CC-3′, 1st reverse 5′-TCR GCA CTC GAG TAC ATC TG-3′ 2nd Forward 5′-CTA GCC GAG TAG CGT TGG G-3′, 2nd Reverse 5′-GCT AGR GTC TTR TTG CCA GC-3′ for 2a, and 1st forward 5′-GCT AGC CGA GTA GTG TTG GG-3′, 1st reverse, 5′-ACC AAR TAA AGG TCC GAG CTG-3′, 2nd Forward, 5′-TCT CGT AGA CCG TGC ACC A-3′, 2nd Reverse 5′-AGA CRG TCC AAC ACA CRC C-3′ for 1b. PCR amplicons (5′UTR to NS3) of each genotype depend on primers design.

Total RNA was extracted from liver tissue samples using Tri-Reagent. RT-PCR and nested PCR reactions were performed with the same primers and enzymes used for sera samples 1b and 2a. PCR products were examined on 1% (w/v) agarose gel stained with ethidium bromide. Amplicons were gel-purified with QIAquick Gel Extraction Kit (QIAGEN), treated with the PCR Clean-Up System (Prom-ga) and subjected to bidirectional Sanger sequence analysis. The PCR-synthesized DNA viral fragments were cloned in the TOPO-TA cloning system (Thermo Fisher Scientific), according the manufacturer’s instructions. Plasmid DNA from the selected bacterial clones was purified by the alkali lysis method and sequenced (CeMIA SA, Larissa, Greece).

### Cells and RNA Transfections

The Huh7.5 hepatoma cell line used was maintained in high glucose Dulbecco’s modified Eagle medium, supplemented with 2 mM glutamine, 10% (v/v) heat-inactivated fetal calf serum, 100 U/ml penicillin/streptomycin and non-essential amino acids. For RNA transfections, the protocol used is essentially described in Lohmann (2009). *In vitro* transcribed RNA from the pFK-I_341_PI-Luc/NS3-3′/JFH1–replicating construct (Steinmann et al., 2008) and RNA constructs that represent full length sequences from 5′UTR to NS3 (FL) or IFDMs HCV genomes were introduced by electroporation into the Huh7.5 cell line at a 1:1 ratio. Huh7.5 cells were also electroporated with 1:1 ratio of JFH1:ΔE1E2 RNA constructs. JFH-1 HCV genotype 2a strain (full length genome) isolated from a patient with fulminant hepatitis, is the only strain capable of replicating efficiently in Huh7 human hepatoma cells. The JFH1/ΔE1E2 construct lacks the E1–E2 envelope region (Wakita and Kato, 2006). Cells were electrotransfected with 10 μg RNA and equal amounts of carrier t-RNA. Following transfection, cells were harvested, seeded into 48-well plates, collected at the appropriate time points and subjected to luciferase assays with a commercially available kit (Promega). Luciferase activity was normalized to protein in order to yield relative luciferase activity (RLA). The reported values were collected from seven independent experiments.

### Exosomes Isolation

Exosomes were isolated from sera samples with the Exosome Precipitation Solution (Macherey-Nagel) according to the manufacture instructions. Exosomal pellets were resuspended in PBS and kept at -80°C until use.

### Western Blotting

For Western blotting analysis exosomes were treated with RIPA Buffer (1.0% (v/v) NP-40 or Triton X-100, 0.5% (w/v) sodium deoxycholate, 0.1% (w/v) SDS, 50 mM Tris, pH 8.0), electrophoretically separated on 10–12% (w/v) SDS-gels, transferred onto nitrocellulose membranes and incubated with appropriate antibodies – rabbit anti-CD63 pAb-H-193, Santa Cruz Biotechnology; rabbit anti-CD9 pAb, Cell Signaling; mouse anti-Hsp90 mAb, kindly provided by Dr. Patsavoudi (Thomaidou and Patsavoudi, 1993). The immunocomplexes were detected by enhanced chemiluminescence (Pierce).

### Measurement of HCV Ag (Core) and HCV RNA

The HCVAg (Core) was measured in cell lysates and supernatants using the Abbott ARCHITECT HCV Ag assay. This is a two-step immunoassay, using Chemiluminescent Microparticle Immunoassay (CMIA) technology, with flexible assay protocols referred to as Chemiflex, for the quantitative determination of HCV core antigen. The concentration of Hepatitis C core antigen in the specimen was determined using a previously generated ARCHITECT HCV Ag calibration curve and a baseline value of 3.00 fmol/L.

The HCV RNA was quantified with the SACACE HCV Real TM Quant PCR kit, using the Mx3000P QPCR System (Stratagene). HCV RNA was extracted from plasma, reverse-transcribed to cDNA and detected using HCV-specific fluorescent probes, according to manufacturer’s instructions.

### Statistical Analysis

Statistical analysis was performed using one-way ANOVA for more than two samples and Student’s *t*-test as a *post hoc* or comparisons between two samples. *p* ≤ 0.05 was considered statistically significant (^∗^*p*-value ≤ 0.05; ^∗∗^*p*-value ≤ 0.005; ^∗∗∗^*p*-value ≤ 0.001). Unless otherwise stated, statistical analysis was carried out between control and treated cells.

## Results

### Isolation and Characterization of HCV Natural Mutants, Containing Large In-frame Deletions

**FIGURE 1:**
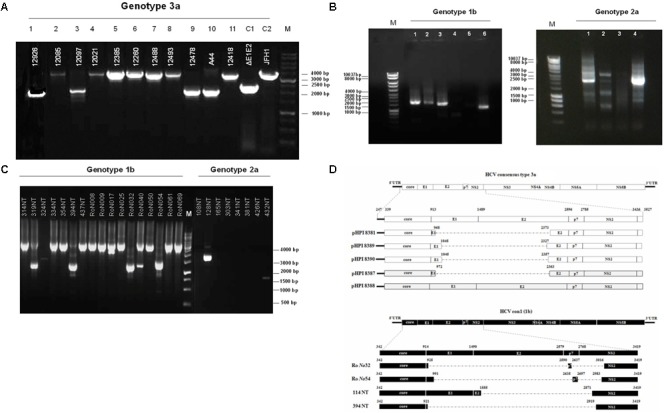
Detection of HCV natural mutants, containing large in-frame deletions in sera samples and liver tissue of HCV-infected individuals with different genotype. **(A)** Nested PCR amplicons from HCV 3a-infected sera samples were examined on 1% (w/v) agarose gel. Lanes 1, 3, 9, 10 correspond to sera samples containing IFDMs. The remaining lines 2, 4, 5, 6, 7, 8, 11 correspond to sera samples containing full length genomes. Positive controls are depicted in lanes C1, JFH1 full length; C2, JFH1 ΔE1E2. Line M, molecular size marker 1 Kb. **(B)** PCR amplicons from 1b and 2a HCV samples were similarly examined, using 1 Kb marker, where the IFDMs were observed on 1, 2, 3, 6 lanes for 1b and on 1, 2, 4 lanes for 2a. **(C)** Nested-PCR amplification products from liver tissue samples of HCV-infected patients (genotypes 1b and 2a). **(D)** Schematic representation of the defective and full length genomes’ architecture identified in the sera samples and liver tissues of HCV patients chronically infected with genotypes 3a and 1b.

Hepatitis C defective genomes detected in serum and liver specimens of HCV patients present large in-frame deletions localized mainly within the structural region of the viral genome from E1 up to NS2 (Yagi et al., 2005; Iwai et al., 2006; Noppornpanth et al., 2007; Sugiyama et al., 2009). HCV DIs have been detected in 18.9% of HCV patients with genotype HCV 1 (Cheroni et al., 2015). In order to test our group of chronic HCV patients for the presence of IFDMs and further investigate their biological role, we isolated viral RNA from sera (genotype 1b, 2a, 3a) and liver tissues (genotype 1b, 2a) of HCV-positive clinical samples. The size of PCR amplicons shown in Figure 1 confirmed the existence of IFDMs in both serum and liver tissues. Specifically, for genotype 3a, results from a representative number of samples revealed the presence of IFDMs of average length around 2100 bp. PCR amplicons representing the FL viral genome of 3200 bp (from 5′UTR to NS3) were also detected, as showed in Figure 1A. Furthermore, the PCR amplicons of JFH1 and JFH1/ΔE1E2 resulting in known size DNA fragments were used as positive controls. Sanger sequencing analysis revealed that the obtained IFDMs always contained a deletion in the E1E2 structural region, thus maintaining the core region preserved. With regard to patients infected with HCV genotypes 1b and 2a, PCR analysis detected a FL sequence of 4000 bp and the presence of IFDMs with different sizes (1500, 2000, and 2500 bp) (Figure 1B). Importantly, HCV sera samples of genotype 3a contain IFDMs that rise up to 26%. For sera samples of genotype 2a and 1b, the percentage reached 20 and 26% correspondingly. Additionally, IFDMs were present in 20 and 8.3% of liver tissue samples from patients infected with HCV genotypes 1b and 2a, respectively (Table 1). Interestingly, IFDMs were detected in the majority of clinical samples with high viremia. Their presence seems independent of the type of sample, either sera or liver tissue.

In order to facilitate the investigation of the biological role of IFDMs, PCR amplicons representing defective or full length viral sequences were cloned in the TOPO-TA cloning system (Thermo Fisher Scientific). The plasmids carrying IFDMs isolated from sera samples 3a are presented as pHPI 8381, 8387, 8389, and 8390. pHPI 8388 expresses the FL sequence of HCV 3a, isolated from sera infected with the corresponding viral strain. Similarly, we generated plasmids RoNo32, RoNo54, 114NT, and 394NT, encoding for IFDMs isolated from liver tissues infected with HCV 1b. Sequence analysis of those plasmids showed that deletions are mostly localized in the structural region (E1E2), as schematically presented in Figure 1D. Since the observed deletions were almost of the same size and localized in the same viral genomic region, we chose to clone IFDMs from the readily available HCV genotypes within our specimen collection, such as HCV 3a (sera) and HCV 1b (liver tissues).

### Effect of IFDMs on Viral Replication

**FIGURE 2:**
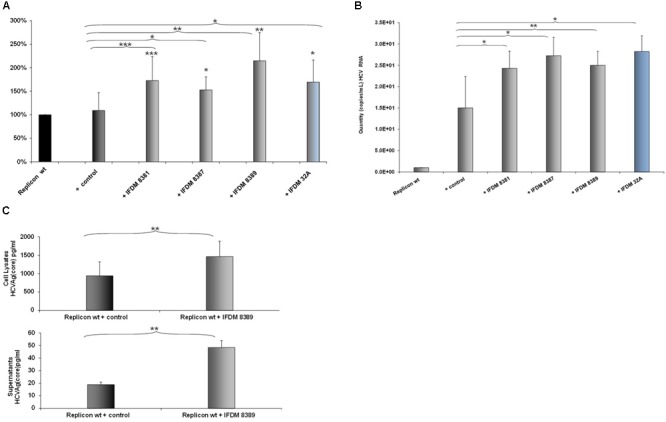
Effect of HCV defective genomes on viral replication and release. **(A)** Effect of IFDMs on HCV replication. The pFK-I_341_PI-Luc/NS3-3′/JFH1–replicating construct (replicon wt) was electroporated in Huh7.5 cells simultaneously with different RNA IFDMs constructs at a ratio 1:1. pHPI 8388 clone (containing the FL HCV sequence) served as a control. The majority of the IFDMs used were HCV genotype 3a (8381, 8387, 8389) and 1b (32A). Cells were collected at 48 h, lyzed and luciferase activity was measured and normalized to total protein. The value for replicon wt was arbitrarily set as 100% and all other values are a percentage of this (seven independent experiments). **(B)** Quantity of viral RNA (viral copies/mL) from replicon wt, control (containing the FL HCV sequence) and IFDMs 8381, 8387, 8389, 32A measured in the cell culture supernatants of the above experiment with the SACACE Real Time PCR kit for HCV detection. **(C)** HCV Ag (Core) was measured in cell lysates and supernatants from replicon wt, control (containing the FL HCV sequence) and IFDM HCV 3a (8389) using the Abbott ARCHITECT HCV Ag assay. ^∗^*p*-value ≤ 0.05; ^∗∗^*p*-value ≤ 0.005; ^∗∗∗^*p*-value ≤ 0.001.

A representative number of clones containing HCV defective and full length RNA genomes (depicted in Figure 1D) isolated from sera samples and liver tissues, were used for *in vitro* RNA production. Each one of these RNAs was co-transfected at a 1:1 ratio with RNA produced from the pFK-I_341_PI-Luc/NS3-3′/JFH1–replicating construct in Huh7.5 cells. 48 h post-electroporation, a time point that signifies the peak of viral replication with the pFK-I_341_PI-Luc/NS3-3′/JFH1–replicating construct, cells were collected and lyzed. Subsequently, luciferase activity was measured. The results (Figure 2A) indicated increased viral replication in the presence of HCV IFDMs. The RNA construct from clone pHPI 8388 (isolated from a serum sample containing the FL HCV genome) served as a control. The majority (70%) of the IFDMs used were HCV genotype 3a (8381, 8387, and 8389) and 1b (32A). The results showed that in the presence of these IFDMs HCV replication was increased by up to twofold, when compared either with the replicon wt alone or with the control RNA. Next, we measured both viral RNA in supernatants alone (Figure 2B) and HCV Ag (Core) in cell lysates and supernatants (Figure 2C) from our transfection experiments. We observed a significant increase of HCV viral RNA (copies/mL) in the presence of IFDMs (Figure 2B). Our results clearly demonstrated that the release of viral RNA was up-regulated in the presence of IFDMs. Determination of HCV Ag (Core) in cell lysates and supernatants showed that core protein was over-expressed in the cell lysates and abundantly released extracellularly by up to 2.5-fold in sample 8389, as compared to the control. The HCV Ag (Core) measured could have originated from a mixed population of infectious viral particles and naked particles. Furthermore, the viral RNA determined in the supernatants could collectively represent the viral genome of infectious viral particles, naked particles and exosomes.

### Modulation of Viral Release in the Presence of HCV IFDMs

**FIGURE 3:**
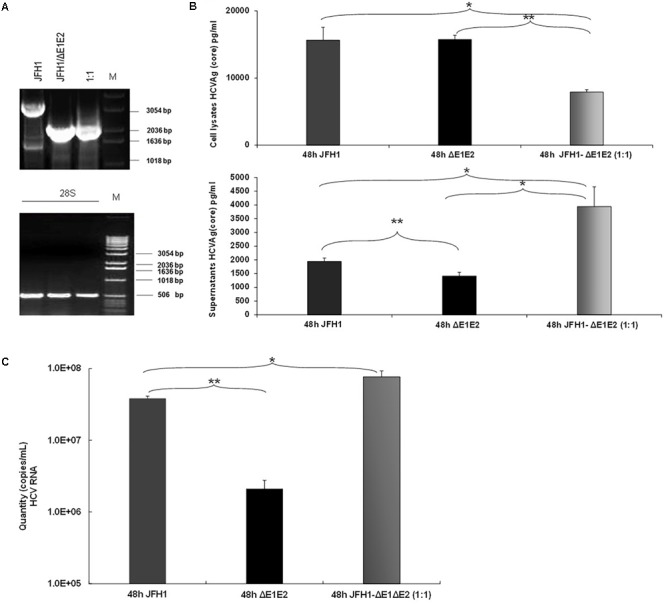
Prevalence of IFDMs in the HCV *in vitro* cell culture system. **(A)** Electroporation experiments were performed in Huh7.5 cells using RNA JFH1 or JFH1 ΔE1E2 at ratios 1:1. The 1% (w/v) electrophoretogram depicts amplicons from isolated IFDMs, following RNA isolation and RT-PCR analysis. M, Molecular size marker. **(B)** HCV Ag (Core) was measured at 48 post-transfection in cell lysates and supernatants using the Abbott ARCHITECT HCV Ag assay. **(C)** Quantity of viral RNA (viral copies/mL) was measured in the above cell culture supernatants using the SACACE Real Time PCR kit for HCV detection. ANOVA analysis showed statistical significance between samples. ^∗^*p*-value ≤ 0.05; ^∗∗^*p*-value ≤ 0.005.

Previous studies demonstrated that the naturally occurring HCV subgenomes are capable of autonomous replication and *trans*-packaging in Huh-7.5 cells infected with wt J6/JFH virus (Pacini et al., 2009). In order to investigate the biological role of HCV natural mutants containing large in frame deletions in viral release, we used the HCV *in vitro* cell culture system. Electroporation experiments were performed in Huh7.5 cells with RNA constructs that carry the HCV full length genome JFH1 or JFH1/ΔE1E2. The corresponding JFH1 and JFH1/ΔE1E2 RNAs were simultaneously electroporated at 1:1 ratio. 48 h post-transfection/electroporation, cells were lyzed and the viral RNA was isolated. Nested RT-PCR was performed in order to detect the presence of IFDMs. As demonstrated in Figure 3A, co-transfection at 1:1 ratio showed that the presence of defective viral genomes predominated over that of full length genomes. The presence of the full length genome at 1:1 ratio was, as expected, less visible than in the wt virus alone. In parallel, HCV Ag (Core) was measured in the cell lysates and culture supernatants (Figure 3B). At 48 h and 1:1 ratio of viral RNAs (JFH1:JFH1/ΔE1E2), the HCV core antigen measured in the cell lysates was significantly decreased with a concurrent up-regulation in the supernatants that did not occur in the absence of E1–E2 in the JFH1/ΔE1E2 construct, as expected. The observed twofold up-regulation of HCV core antigen measured in the supernatants of JFH1:JFH1/ΔE1E2 was statistically significant relatively to the corresponding JFH1. Moreover, HCV viral RNA (copies/mL) measured in the same supernatants was found to be significantly increased (Figure 3C). Taken together, these data indicate that HCV particles are formed and viral release is increased when both full length and defective forms are present. In our previous studies (Karamichali et al., 2017), we have shown that in the presence of HCVne (hepatitis C non-enveloped capsid-like particles) viral production was significantly increased by 2.5-fold. With the current experiment, we propose that the non-enveloped HCV particles produced by the JFH1/ΔE1E2 construct, may enhance viral release from host cells, in the presence of the wt virus.

### Exosomes Mediate Transfer of IFDMs Thus Regulating Viral Replication

Exosomes are considered intercellular transporters of cellular components such as RNA, lipids, and proteins. Over the last years, several studies indicated that these organelles are capable of transferring viral components. It has been reported that HCV genome can be intercellularly transmitted via exosomes (Dreux et al., 2012; Ramakrishnaiah et al., 2013; Liu et al., 2014). Therefore, it would be of interest to investigate whether this mechanism applies to IFDMs and outline a new role for exosomes in HCV persistence.

**FIGURE 4:**
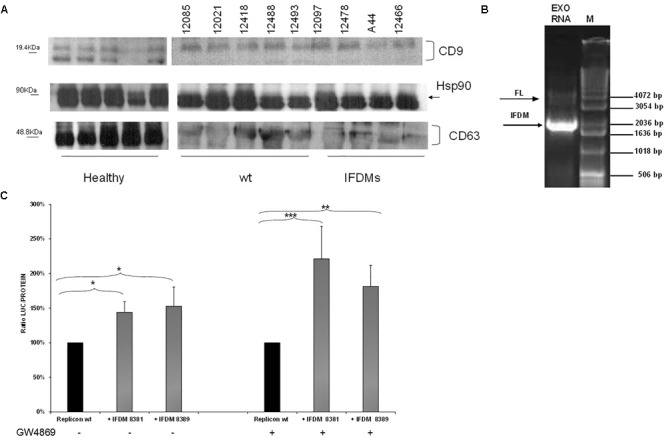
Identification and function of exosomal IFDMs. **(A)** Exosomes were isolated, with the Exosome Precipitation Solution (serum plasma; Macherey-Nagel), from sera samples of three distinct groups, healthy blood donors (Healthy), chronic HCV patients with full length genomes (wt) and HCV patients with IFDMs of genotype 3a (IFDMs). Exosomes were characterized by Western blotting analysis using antibodies against exosomal markers CD63, CD9 and Hsp90. **(B)** In a representative experiment, exosomal RNA (Exo RNA) was subjected to nested PCR analysis and run on a 1% (w/v) agarose gel against a molecular size marker (M). Bold and thin arrows indicate the IFDM and FL sequences, respectively. **(C)** pFK-I_341_PI-Luc/NS3-3′/JFH1-replicating construct was co-electroporated with representative IFDMs RNA constructs in Huh7.5 cells in the presence of the exosomal inhibitor GW4869. 5 μM of the inhibitor GW4869 (Sigma) were added to the electroporated cells, in order to block exosomes’ release. Cells were collected at 48 h, lyzed and luciferase was measured and normalized to total protein. The values for replicon wt/replicon wt+inhibitor were arbitrarily set as 100% and all other values are a percentage of these. ^∗^*p*-value ≤ 0.05; ^∗∗^*p*-value ≤ 0.005; ^∗∗∗^*p*-value ≤ 0.001.

For this purpose, sera samples from three different groups (healthy, patients with full length (wt) and IFDMs genomes of HCV 3a) were used to isolate exosomes. Characterization of exosomes was performed by western blotting using antibodies against the typical exosomal markers CD63, CD9, and Hsp90 (Figure 4A). CD9 and CD63 exosomal markers were differentially expressed between healthy donors and infected samples. For example, CD63 glycosylation status was reduced in both the infected groups. These changes are currently being investigated in our laboratory in order to clarify their role in exosome-mediated transfer of IFDMs. Viral RNA extracted from exosomes showed the presence of IFDMs (Figure 4B), suggesting that HCV defective genomes can be transferred within exosomes.

Since we have previously demonstrated that the presence of HCV IFDMs up-regulated viral replication, we then decided to investigate whether blocking exosomal release from cells could modulate this phenomenon. RNA from pFK-I_341_PI-Luc/NS3-3′/JFH1–replicating construct was electroporated with representative RNA IFDMs at 1:1 ratio in Huh7.5 cells in the presence or absence of the exosomal inhibitor GW4869 (Sigma, 5 μM). The inhibitor blocks both exosome biogenesis and cellular release. Cells were collected at 48 h, lyzed and luciferase activity was measured and normalized against total protein. Surprisingly, as showed in Figure 4C, in the presence of exosomal inhibitor viral replication was further up-regulated by up to 1.5-fold. In this experiment we observed similar accumulation of core protein in the cell lysates in the presence or absence of the inhibitor (Supplementary Figure 1). Furthermore, the inhibitor GW4869 did not affect pFK-I_341_PI-Luc/NS3-3′/JFH1 replication because luciferase activity was similar in its presence or absence (data not shown). Therefore, the observed excessive effect on viral replication could be attributed to synergistic action between the inhibition of exosomal release and the accumulation of over-expressed core. In our previous studies (Karamichali et al., 2017), we have shown that the presence of the particulate form of HCV core was able to up-regulate viral replication. In the present study, we demonstrate that HCV replication is also up-regulated in the presence of IFDMs, where core is over-expressed. Therefore, it is possible that the combination of exosome-mediated viral RNA release blockage and core overexpression could lead to more pronounced HCV replication.

## Discussion

In this study, we demonstrated that IFDMs, lacking the envelope region, are present in sera samples taken from chronic HCV patients infected with HCV genotypes 1b, 2a, and 3a, at percentages ranging between 20 and 26%. We have used sera samples and liver tissues of different HCV genotypes in order to investigate whether there is any correlation between the presence of IFDMs and the sampling origin. Our results demonstrated that either in sera samples or liver tissues of genotype 1b IFDMs, percentages did not differ. An 8.3% IFDM prevalence in liver samples from HCV 2a-infected individuals was attributed to issues with PCR amplification and most probably does not reflect the true levels of IFDMs present in this HCV genotype. Furthermore, we did not observe significant differences in the percentage of IFDMs between the three HCV genotypes tested. The only common parameter between all clinical samples containing IFDMs was high viremia (data not shown). Thus, the sustained presence of IFDMs, unexplored until now, independently of sampling origin and HCV genotype, raises questions about their biological significance in the HCV life cycle and carries connotations about their involvement in HCV persistent infection.

Pacini et al. (2009) reported that subgenomic deletions are replication-competent and that there is interplay between core and NS2 regions in the regulation of *trans*-packaging and possibly RNA replication. In an attempt to further investigate these arguments, we used a twofold approach, with cloned IFDMs from clinical samples infected with HCV 3a and an IFDM derived from the HCV *in vitro* cell culture system (JFH1/ΔE1E2), in electroporation experiments together with the JFH1 HCV full length genome or the replicon construct. We observed for the first time that in the presence of IFDMs originating from clinical samples, HCV viral replication was increased. Thus, coexistence of defective and full length genome of the virus may lead to increased replication.

Subsequently, electroporation experiments performed in Huh7.5 cells using RNA constructs carrying the HCV full-length genome JFH1 or JFH1/ΔE1E2, showed that in the presence of defective genomes the HCV core antigen measurement in the supernatants was elevated. This result, indicates that the virus’s improved release from the host cell will potentially lead to more efficient viral spread.

This phenomenon led us to hypothesize that *trans*-complementation events can occur. Ke et al. (2013) have shown that defective Dengue virus is not only co-transmitted with the wt virus, but also increases transmission of the functional viral particles by at least 10%. Moreover, they suggested that coexistence of defective and functional virus in the same cell makes the functional virus replicate better within the cell through unknown mechanisms (Ke et al., 2013), thus increasing viral disease transmission. As recently stated, although decades of work on defective viral genomes and their functions have been done, much remains unknown. There are different types of defective viral genomes, therefore, it is conceivable that immunostimulation, interference and persistence exist as separate functions for at least some. It is possible that persistently infected cells constantly swap between defective and full length viral genomes, depending on viral life cycle needs (Manzoni and Lopez, 2018).

Exosomes are implicated in cell communication transferring double-stranded DNA, mRNAs, microRNAs (miRNAs), and long noncoding RNAs (lncRNAs), to recipient cells modulating their functions (Schorey et al., 2015). It has been reported that in the context of the HCV infection, exosomes transfer the full length viral HCV RNA and mediate HCV release from infected hepatocytes (Shrivastava et al., 2015). We showed for the first time the presence of IFDMs lacking the envelope region within exosomes. The use of a compound that inhibits biogenesis of exosomes and prevents their release from the cells, offered an unexpected additional increase in viral replication, unlike the one initially observed in the absence of the exosomal inhibitor. At the same time, we observed an overexpression of core protein in the cell lysates and its subsequent release in the culture supernatants. An intriguing explanation for these phenomena may be that the exosomes’ release inhibition mechanism and the intracellular accumulation of core protein may synergistically modulate viral release from the host cells leading to increased HCV replication. Support for this hypothesis comes from our previous studies (Karamichali et al., 2017), which showed that the particulate form of core protein up-regulates HCV replication, indicating a new functional role for the core protein in the HCV life cycle. Furthermore, the IFDMs that have been isolated from sera samples and liver tissues of HCV-infected individuals present mainly with a deletion in the structural region E1E2, thus maintaining the core region preserved and indicating an important role for the core protein. It has been mentioned that core protein accumulation could probably be a consequence of the efficient replication of IFDMs and the lack of viral egress could influence and contribute to liver pathogenesis during HCV-associated chronic hepatitis progression (Pacini et al., 2009). Moreover, another study reports characteristic core accumulation in cells infected with defective HCV genomes, as opposed to cells infected with wt virus, where excess core is released in the form of mature viral particles (Cheroni et al., 2015).

Defective interfering viruses may play a role in the establishment and maintenance of persistent infection. Specifically, Flaviviruses have been reported to use DI genomes to establish chronic infections (Lancaster et al., 1998; Yoon et al., 2006). We demonstrated that in the presence of IFDM RNAs, viral replication and release are up-regulated. In addition, we observed that IFDMs can be transferred through exosomes and that their blockage in the cell may elevate viral replication further, potentially establishing an HCV persistent infection. The notion that HCV particles carrying defective genomes may act as “immunological decoys” and divert immune responses away from the wt virus, has been proposed by Cheroni et al. (2015). Evidently, such a mechanism could favor viral persistence over viral clearance.

## Conclusion

The detection of HCV defective genomes in clinical samples infected with different HCV genotypes led us to the investigation of their distinct biological significance. We show herein that in the presence of HCV IFDMs the viral replication as well as the virus release are increased. Importantly, we propose a novel role for exosomes as carriers of defective viral genomes and as means for the virus to avoid the immune system and spread more efficiently, with possible implications on viral persistence.

## Author Contributions

EK and UG conceived, and designed the experiments and analyzed the data. EK, HC, AgM, TK, and AK performed the experiments. EK, UG, AK, HC, SB, AM, VP, AnK, AV, PP, BM-G, and IK contributed to the reagents, materials, and analysis tools. EK, UG, and PF wrote the article.

## Conflict of Interest Statement

The authors declare that the research was conducted in the absence of any commercial or financial relationships that could be construed as a potential conflict of interest.

## Supplementary Material

FIGURE S1 pFK-I341PI-Luc/NS3-3′/JFH1-replicating construct was co-electroporated with representative IFDMs RNA constructs in Huh7.5 cells in the presence or absence of the exosomal inhibitor GW4869. HCV Ag (core) was measured in the cell lysates at 48 h post electroporation.

## Abbreviations

DI: defective interfering
FL: full length HCV sequence from 5′UTR to NS3
IFDMs: in-frame deletions mutants
wt: wild type
