# Supplementary figures and images for: HCV Defective Genomes Promote Persistent Infection by Modulating the Viral Life Cycle
Source: Front Microbiol. 2018 Dec 3;9:2942. doi: 10.3389/fmicb.2018.02942 (PMC6287115; doi:10.3389/fmicb.2018.02942)

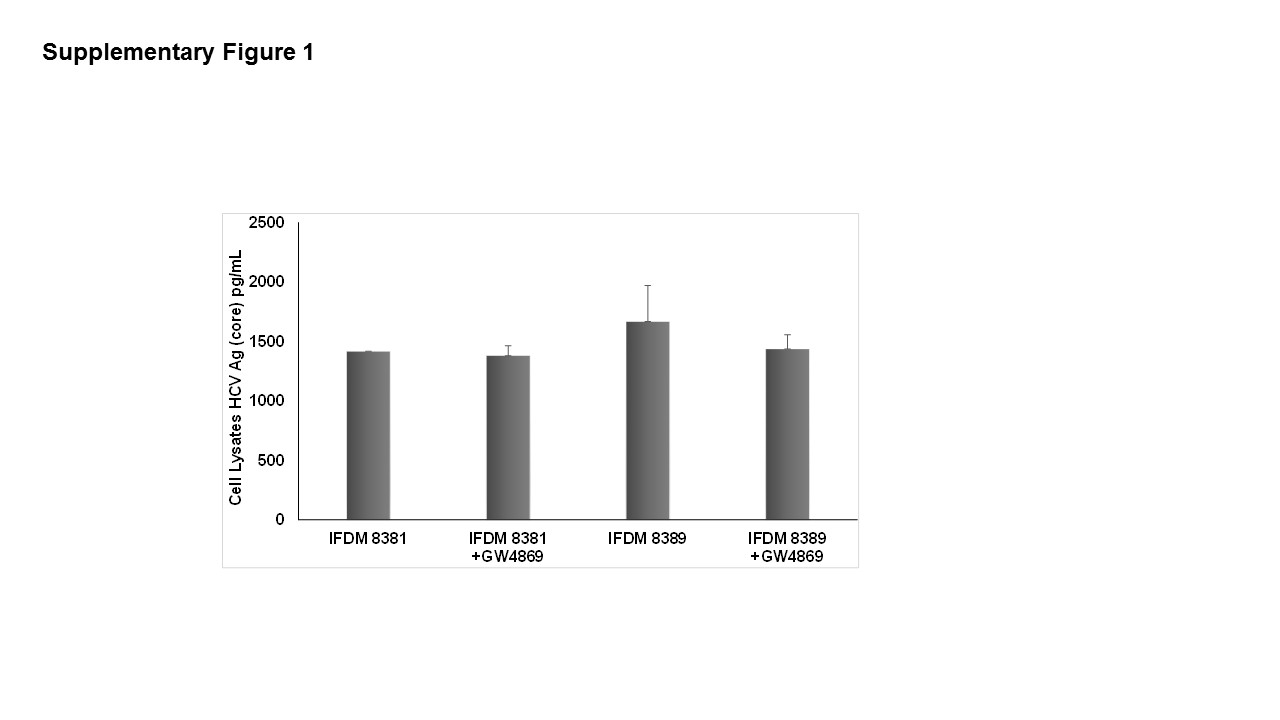

Supplement: FIGURE S1 — pFK-I341PI-Luc/NS3-3′/JFH1-replicating construct was co-electroporated with representative IFDMs RNA constructs in Huh7.5 cells in the presence or absence of the exosomal inhibitor GW4869. HCV Ag (core) was measured in the cell lysates at 48 h post electroporation. [file Image_1.JPEG]
